# Integrated Assessment of Hematoma Burden, Anatomical Extension, and Mass Effect for 90-Day Functional Outcome After Spontaneous Supratentorial Intracerebral Hemorrhage: A Temporal Validation Study

**DOI:** 10.3390/diagnostics16213684

**Published:** 2026-11-07

**Authors:** Semih Kivanc Olguner, Mustafa Emre Sarac

**Affiliations:** Department of Neurosurgery, Adana City Training and Research Hospital, Adana 01230, Turkey; emre_sarac@hotmail.com

**Keywords:** cerebral hemorrhage, hematoma volume, midline shift, cerebral intraventricular hemorrhage, prognosis, neuroimaging

## Abstract

**Objective:** To assess the contribution of hematoma burden, anatomical extension, and mass effect to predicting 90-day poor functional outcome alongside clinical characteristics in spontaneous supratentorial intracerebral hemorrhage, and to evaluate temporal model performance. **Methods:** This single-center retrospective study included 789 patients undergoing CT within six hours of symptom onset: 508 in the development cohort (2018–2022) and 281 in the temporal validation cohort (2023–2025). Poor outcome was defined as a 90-day modified Rankin Scale score of 4–6. Hematoma volume, deep structure involvement, and intraventricular hemorrhage were added to clinical logistic regression models, followed by midline shift. Discrimination and calibration were assessed, with 1000 bootstrap resamples for internal validation. Model coefficients remained unchanged during temporal validation. Secondary analysis replaced absolute shift with the shift/volume ratio while retaining hematoma volume. **Results:** Poor outcome occurred in 46.1% and 44.5% of patients, respectively. Development AUC increased from 0.798 to 0.846 with volume and extension variables (*p* < 0.001), then to 0.875 with midline shift (*p* = 0.014). Hematoma volume, intraventricular hemorrhage, and midline shift remained associated with poor outcome. The adjusted odds ratio per millimeter of shift was 1.17 (95% CI, 1.08–1.28). Optimism-corrected and temporal validation AUCs were 0.856 and 0.854. Model ranking persisted, with generally concordant predicted and observed risks. The association between midline shift and poor outcome persisted in sensitivity analyses addressing surgery or external ventricular drainage, care limitations, and CT timing. The ratio was associated with poor outcome but yielded numerically lower AUCs in both cohorts. **Conclusions:** The model incorporating all three radiological features showed the highest discrimination among the three models in both cohorts, supporting joint assessment of hematoma burden and mass effect. Multicenter validation and clinical utility assessment remain necessary.

## 1. Introduction

Spontaneous intracerebral hemorrhage (ICH) is a major cause of stroke associated with high mortality and lasting functional impairment. Recent data indicate that approximately one-third of patients die within the first month, and only about one-third achieve a favorable functional outcome at 3–12 months [1]. Standardized acute care and minimally invasive surgery in selected patients can improve functional outcomes [2,3]. Prognostic assessment based on admission data is important for assessing disease severity and discussing prognosis with patients’ families [4,5].

Prognosis after ICH is associated with clinical and radiological characteristics, including age, level of consciousness, hematoma volume, hemorrhage location, and ventricular extension. Existing prognostic scores combine these variables to predict mortality or functional recovery [6,7]. Coexisting cerebral small vessel disease and the anatomical location of hemorrhage are also associated with clinical outcomes [8,9]. These findings support considering both the patient’s clinical condition and the structures affected by the hemorrhage when assessing functional prognosis.

Admission computed tomography (CT) allows the assessment of hematoma volume, extension, and effects on surrounding structures [9]. Although hematoma volume is a central measure of hemorrhage burden, the volumes associated with poor outcomes differ across anatomical regions, supporting interpretation of volume alongside location [10]. Associations of hematoma expansion and increasing perihematomal edema with poor outcomes suggest that injury after ICH is not limited to the initial hematoma volume [11,12]. Midline shift (MLS) reflects displacement of brain structures and provides information about mass effect. Its association with three-month functional outcome on admission CT supports its relevance to early prognostic assessment [13,14]. Midline shift at the end of treatment and changes in shift over time have also been associated with clinical outcomes [15].

Recent studies have assessed radiomic features of parenchymal and intraventricular hemorrhage (IVH) to predict hematoma expansion [16], and features of the hematoma and perihematomal edema combined with clinical data to predict three-month functional outcome [17]. In routine practice, hematoma volume, anatomical extension, and MLS provide prognostic information available from admission CT. Their associations with outcome alone do not establish how much they improve prediction when combined with clinical data. Assessing the additional contribution of MLS when hematoma volume and extension are already considered may clarify the role of mass effect in this assessment. Evaluation in subsequent patients also allows predictive performance to be assessed beyond the development cohort [18].

This study aimed to assess the contribution of hematoma burden, anatomical extension, and mass effect, considered alongside clinical characteristics, to predicting 90-day poor functional outcome in patients with spontaneous supratentorial ICH. Radiological features were added sequentially to the clinical model, and predictive performance was assessed through internal and temporal validation. A secondary analysis examined whether expressing MLS relative to hematoma volume changed predictive performance compared with including volume and absolute shift as separate variables.

## 2. Methods

### 2.1. Study Design and Patient Selection

This single-center retrospective cohort study was designed to develop a prognostic model combining clinical characteristics, hematoma burden, anatomical extension, and MLS in adults with primary spontaneous supratentorial ICH and to evaluate its performance in a temporally separate cohort. The study was conducted at a tertiary referral center providing both neurosurgical and neurocritical care services. Clinical and imaging data collected during routine care were reviewed retrospectively. The development cohort comprised patients admitted between 1 January 2018 and 31 December 2022, and the temporal validation cohort comprised those admitted between 1 January 2023 and 31 December 2025. Prognostic models were developed in the development cohort and evaluated in the temporal validation cohort.

Hospital records were reviewed to identify all patients admitted with ICH during the study periods. A total of 1459 patients were screened: 938 during the development period and 521 during the temporal validation period. Patients were eligible if they were aged 18 years or older, had a documented diagnosis of primary spontaneous supratentorial ICH, underwent non-contrast head CT at our center within six hours of symptom onset, and had an admission Glasgow Coma Scale (GCS) score recorded before sedation and endotracheal intubation.

Hemorrhage etiology was assessed by reviewing clinical records and available imaging findings. In addition to the initial head CT, this assessment used CT angiography, brain magnetic resonance imaging, magnetic resonance angiography, and digital subtraction angiography when performed as part of individual patient care. Use of the same advanced imaging modality in all patients was not required for inclusion. For patients who died early, etiological assessment was based on clinical and imaging data obtained before death; incomplete advanced imaging alone was not a reason for exclusion. Patients were excluded if the available evaluation identified traumatic intracranial hemorrhage or hemorrhage attributable to an aneurysm, arteriovenous malformation, cavernous malformation, intracranial tumor, cerebral venous thrombosis, or hemorrhagic transformation of an ischemic stroke. Additional exclusions were infratentorial hematomas, initial CT performed at another institution, absence of a qualifying admission GCS assessment before sedation and endotracheal intubation, and image quality insufficient for reliable radiological measurements.

Symptom onset was determined from emergency department records and the clinical history documented in the medical record. Onset was defined as the time neurological symptoms were first noticed by the patient, a family member, or a healthcare professional. When the exact onset time could not be established, the last known well time was used as the reference for the six-hour eligibility window. Patients were excluded if the interval from the applicable reference time to CT exceeded six hours or if the available timing information was insufficient to confirm eligibility.

Exclusion criteria were applied sequentially in the order shown in Figure 1, with each excluded patient counted once under the first applicable criterion. Eligibility screening excluded 409 patients from the development period and 232 from the temporal validation period. These criteria identified 529 patients in the development cohort and 289 in the temporal validation cohort. Of these, 21 and eight patients, respectively, were excluded because at least one clinical model variable or the 90-day functional outcome was missing. The final study population comprised 789 patients: 508 in the development cohort and 281 in the temporal validation cohort. The proportions of patients with missing data were 4.0% and 2.8%, respectively. The primary analyses used a complete-case approach, including patients with complete data for all model variables and the 90-day functional outcome. Figure 1 presents the initial screening populations, exclusions by reason, and final cohort sizes separately for the two study periods.

### 2.2. Clinical Variables and Data Collection

Clinical data were retrieved retrospectively from the hospital information system and electronic medical records. Recorded variables were age, sex, admission GCS score, systolic blood pressure, serum glucose, international normalized ratio (INR), and anticoagulant use before admission. The admission GCS score was obtained from the first neurological assessment in the emergency department before administration of sedative medication and endotracheal intubation. The first systolic blood pressure measurement recorded on arrival was used. Serum glucose and INR values were taken from the first blood sample obtained after admission. Systolic blood pressure was recorded in mmHg and serum glucose in mg/dL. Patients with documented regular use of a vitamin K antagonist or a direct oral anticoagulant before admission were classified as anticoagulant users.

Treatment during the index hospitalization was determined from electronic procedure records, operative reports, and discharge summaries. Recorded procedures included craniotomy for hematoma evacuation, decompressive craniectomy with or without hematoma evacuation, minimally invasive hematoma evacuation, and external ventricular drainage (EVD). EVD was recorded separately, including when performed alongside hematoma surgery. Patients undergoing more than one procedure were identified, and those undergoing none of these procedures were classified as receiving conservative management.

Physician notes and treatment orders were reviewed for documented limitations of care, including decisions not to attempt resuscitation, to withhold or not escalate life-sustaining treatment, or to withdraw life-sustaining treatment. Patients were classified according to the first documented care-limitation decision. The interval from admission to this decision was categorized as ≤24 h, >24–48 h, or >48 h; cases with unavailable timing were recorded separately. Resuscitation decisions were distinguished from limitations of other treatments. Conservative management alone was not classified as a limitation of care. Records with insufficient information to establish limitation status were classified as indeterminate.

### 2.3. Radiological Assessment and Parameter Definitions

Head CT images were reviewed retrospectively. All scans were analyzed in DICOM format, and measurements were performed using RadiAnt DICOM Viewer (version 2024.1; Medixant, Poznań, Poland). Radiological assessments were performed by a neurosurgeon with 12 years of neurosurgical experience who was blinded to 90-day functional outcomes. CT acquisition dates and times were obtained from the picture archiving and communication system (PACS). The interval between symptom onset and CT acquisition was calculated using the last known well time when the exact onset time was uncertain. This interval was recorded in minutes, and the reference used for its calculation was classified as exact symptom onset or last known well time. Quantitative CT assessment was limited to the first non-contrast head CT obtained at our center in patients for whom this interval was six hours or less. Intracerebral hematoma volume was calculated using the ABC/2 method. A was the longest hematoma diameter on the axial slice with the largest hematoma cross-section; B was the greatest diameter perpendicular to A on the same slice; and C was the number of slices containing the hematoma multiplied by slice thickness. Hematoma volume was recorded in milliliters. Midline shift was measured at the level of the septum pellucidum as the maximum perpendicular distance between the anatomical midline and the septum pellucidum and was recorded in millimeters. Anatomical distribution was assessed in terms of lobar location, deep structure involvement, and intraventricular extension. Hematomas confined to the cortex or subcortical white matter without extension into the basal ganglia or thalamus were classified as lobar. Hematoma involvement of the basal ganglia or thalamus was classified as deep structure involvement, regardless of accompanying lobar extension. Intraventricular hemorrhage was defined as hyperdense blood within the ventricular system and recorded as present or absent. In the combined radiological assessment, hematoma volume represented hematoma burden, deep structure involvement and IVH represented anatomical extension, and MLS represented mass effect. As a secondary radiological parameter, the MLS/ICH volume ratio was calculated by dividing MLS in millimeters by hematoma volume in milliliters and expressed in mm/mL. The use of vascular imaging during the index hospitalization was determined from PACS and radiology reports. CT angiography, magnetic resonance angiography, and digital subtraction angiography were recorded separately. The number of patients undergoing at least one of these examinations was also recorded, with each patient counted once in the overall vascular imaging total. Representative non-contrast head CT images illustrating differences in hematoma burden, anatomical extension, and mass effect are presented in Figure 2.

### 2.4. Outcome Assessment

The primary outcome was poor functional outcome 90 days after admission. Functional status was assessed using the modified Rankin Scale (mRS), with scores of 0–3 classified as favorable and 4–6 as poor. The 90-day mRS score was primarily obtained from scores explicitly documented during outpatient follow-up or inpatient assessment within 90 ± 14 days after admission. When multiple assessments were available within this window, the assessment closest to day 90 was used. The same assessment window was applied to patients who remained hospitalized. Patients with documented death within 90 days after admission were assigned an mRS score of 6, regardless of whether another functional assessment was available. For patients without a documented mRS score within the specified window, two investigators independently assigned scores retrospectively using neurological examination findings and follow-up notes describing activities of daily living and dependence during the same period. Disagreements were resolved by jointly reviewing the records and reaching consensus. These patients were classified as having a record-based mRS assessment, and a sensitivity analysis was performed after excluding them. Thirty-day mortality was obtained from the hospital information system and electronic medical records and reported as a descriptive clinical outcome.

### 2.5. Statistical Analysis

Statistical analyses were performed using IBM SPSS Statistics (version 26.0; IBM Corp., Armonk, NY, USA) and R (version 4.3.2; R Foundation for Statistical Computing, Vienna, Austria). Distributions of continuous variables were assessed using histograms and Q–Q plots. Normally distributed variables were presented as mean ± standard deviation, and other continuous variables as median and interquartile range. Categorical variables were expressed as counts and percentages. Patients with favorable and poor functional outcomes were compared separately within each cohort. Continuous variables were compared using the independent-samples *t*-test or Mann–Whitney U test, and categorical variables using Pearson’s chi-square test or Fisher’s exact test. Treatment procedures, care limitations, vascular imaging, and use of last known well time were summarized by cohort. Onset-to-CT times were reported for each cohort and outcome group. Available baseline characteristics of included and excluded patients were compared within each study period using the same tests, with the number of available observations reported for each variable. Among eligible patients, the proportion of patients with missing data was below 5% in both cohorts. Complete-case analyses included patients with all required clinical and radiological variables and a 90-day mRS score available from direct clinical assessment or follow-up records. Three sequential multivariable logistic regression models were fitted in the development cohort to predict 90-day poor functional outcome. Model 1 included age, sex, admission GCS score, systolic blood pressure, serum glucose, INR, and anticoagulant use before admission. Model 2 added hematoma volume, deep structure involvement, and IVH to Model 1. Model 3 further added absolute MLS. Variables were selected based on clinical relevance and entered simultaneously into their respective models. Age, GCS score, systolic blood pressure, serum glucose, INR, hematoma volume, and absolute MLS were treated as continuous variables; the remaining model variables were categorical. The assumption of a linear relationship between continuous predictors and the logit of the outcome probability was assessed using the Box–Tidwell test, and multicollinearity was assessed using the variance inflation factor. Regression results were reported as adjusted odds ratios with 95% confidence intervals. Odds ratios were expressed per 10-year increase in age, 1-point increase in GCS score, 10-mmHg increase in systolic blood pressure, 10 mg/dL increase in serum glucose, 0.1-unit increase in INR, 10 mL increase in hematoma volume, and 1 mm increase in absolute MLS. Discrimination was assessed using the area under the receiver operating characteristic curve (AUC), with approximate 95% confidence intervals calculated using the Hanley–McNeil method. In the development cohort, the incremental contribution of radiological variables was assessed by comparing Models 1 and 2, and that of absolute MLS by comparing Models 2 and 3. Changes in fit between nested models were assessed using the likelihood ratio test, and differences between AUCs obtained in the same patients were assessed using the DeLong test. Agreement between the probabilities predicted by Model 3 and observed outcomes was assessed using calibration plots, the calibration slope, and the calibration intercept. Overall prediction error was measured using the Brier score. Performance calculated in the cohort used to develop the models was reported as apparent performance. Internal validation was performed using 1000 bootstrap resamples from the development cohort. Models were refitted in each bootstrap sample using the same variables and model structure. Optimism was estimated as the mean difference between the AUCs of these models in the bootstrap samples and the original development cohort. Mean optimism was subtracted from the apparent AUCs to obtain optimism-corrected AUCs. Bootstrap methods were used to calculate 95% confidence intervals for calibration curves in both cohorts. During this procedure in the temporal validation cohort, the coefficients estimated in the development cohort were held fixed. The equations for Models 1, 2, and 3 were applied to the temporal validation cohort without coefficient re-estimation or recalibration. AUCs with 95% confidence intervals were calculated for all three models in this cohort, together with calibration measures and the Brier score for Model 3. The MLS/ICH volume ratio was examined in a secondary analysis as an alternative radiological parameter to absolute MLS. For this analysis, the ratio was added to Model 2; absolute MLS and the ratio were not included in the same model. The adjusted odds ratio for the ratio was reported per 0.1 mm/mL increase. The secondary model was developed in the development cohort and applied to the temporal validation cohort with all coefficients, including the intercept, unchanged and without coefficient re-estimation or recalibration. Its discrimination was assessed in both cohorts using AUCs with 95% confidence intervals. To examine the potential influence of record-based mRS assessment, a sensitivity analysis excluded patients whose 90-day mRS scores had been assigned retrospectively from follow-up records. Models 1, 2, and 3 were refitted in the remaining development cohort using the same variables and model structure. The resulting equations were applied to the remaining temporal validation cohort without coefficient re-estimation or recalibration. AUCs in both cohorts and the adjusted odds ratio for absolute MLS from Model 3 in the reduced development cohort were compared with the primary analysis results. Additional sensitivity analyses excluded patients who underwent surgery or EVD, patients with documented care limitations, and both groups together. Patients with indeterminate care-limitation status were also excluded from the second and combined analyses. Models 1–3 were refitted in each development subset and applied unchanged to the corresponding validation subset. Sample sizes, poor outcome counts, AUCs, the AUC difference between Models 2 and 3, and the adjusted odds ratio for MLS were reported. In a separate analysis, onset-to-CT time was added to all three models as a continuous variable per 60 min increase. These models were fitted in the full development cohort and applied unchanged to the validation cohort to reassess the contribution of MLS after accounting for CT timing. Thirty-day mortality was presented as counts and percentages to describe clinical outcomes in each cohort. All statistical tests were two-sided, and *p* < 0.05 was considered statistically significant.

### 2.6. Ethical Approval and Informed Consent Statement

This study was conducted in accordance with the Declaration of Helsinki and approved by the Scientific Research Ethics Committee of Adana City Training and Research Hospital (Meeting No. 25; Decision No. 1301; approval date: 30 April 2026). The committee waived the requirement for additional study-specific informed consent because of the retrospective design. Written informed consent for the use of anonymized clinical data in research had been routinely obtained from all patients or their legal representatives in accordance with institutional regulations and standard clinical practice.

## 3. Results

### 3.1. Study Population and Baseline Clinical and Radiological Characteristics

The final analysis included 789 patients: 508 in the development cohort and 281 in the temporal validation cohort. Poor functional outcome (mRS 4–6) occurred in 234 patients (46.1%) in the development cohort and 125 patients (44.5%) in the temporal validation cohort. Thirty-day mortality rates were 24.8% and 23.1%, respectively. Median onset-to-CT intervals were 85 and 80 min, respectively. Approximately 30% of patients in each cohort underwent surgery or EVD, while the remainder received conservative management. Documented care limitations were identified in 12 (2.4%) and 6 (2.1%) patients; limitation status was indeterminate in 24 (4.7%) and 10 (3.6%), respectively. Decisions not to attempt resuscitation were the most frequent first documented limitation, followed by withholding or non-escalation and withdrawal of life-sustaining treatment. Among decisions with known timing, the >48 h interval was the most frequent category in both cohorts. Demographic, clinical, and radiological characteristics, treatment details, care-limitation status and decision timing, vascular imaging, and outcomes of the study cohorts are presented in Table 1, Panel A.

Transfer from another institution and intubation before arrival were more common among excluded patients. GCS scores were also lower in excluded patients when a pre-sedation assessment was available. Age and sex did not differ significantly between included and excluded patients in either study period. Excluded patients had higher 30-day mortality in the development period, but this difference was not statistically significant in the validation period. Table 1, Panel B presents these comparisons.

### 3.2. Clinical and Radiological Characteristics According to 90-Day Functional Outcome

In both cohorts, patients with poor functional outcomes were older and had lower admission GCS scores than those with favorable outcomes. Serum glucose levels were also higher in the poor outcome groups. Neither sex nor anticoagulant use differed significantly between outcome groups in either cohort. Admission systolic blood pressure and INR did not differ significantly between outcome groups in the development cohort but were higher in the poor outcome group in the temporal validation cohort (*p* = 0.031 and *p* = 0.046, respectively). The onset-to-CT interval was shorter in patients with poor functional outcomes in both the development and temporal validation cohorts (*p* = 0.023 and *p* = 0.041, respectively). On radiological assessment, patients with poor functional outcomes had larger hematoma volumes, greater MLS, and more frequent IVH. Deep structure involvement was numerically more frequent in the poor outcome groups, although the difference was not statistically significant in either cohort. The secondary radiological parameter, the MLS/ICH volume ratio, was also higher in the poor outcome groups in both cohorts. Clinical and radiological characteristics and onset-to-CT intervals according to 90-day functional outcome in the development and temporal validation cohorts are presented in Table 2.

### 3.3. Sequential Multivariable Logistic Regression Analysis

The joint test of nonlinear terms in the Box–Tidwell analysis showed no significant departure from linearity on the logit scale (*p* = 0.38). The highest variance inflation factor (VIF) was 2.6, with no evidence of substantial multicollinearity. In the development cohort, older age, lower admission GCS score, and higher serum glucose were independently associated with 90-day poor functional outcome in Model 1, which included only clinical variables. These clinical variables retained their independent associations in Model 2 after the addition of hematoma volume and indicators of anatomical extension. Among the radiological variables, larger hematoma volume and intraventricular hemorrhage were independently associated with poor functional outcome, whereas deep structure involvement showed no significant association. In Model 3, which additionally included absolute MLS, each 1 mm increase in shift was associated with 90-day poor functional outcome after adjustment for the other variables (adjusted OR, 1.17; 95% CI, 1.08–1.28; *p* < 0.001). Older age, lower GCS score, higher serum glucose, larger hematoma volume, and intraventricular hemorrhage also retained their independent associations in the final model.

Detailed results for all three models are presented in Table 3.

### 3.4. Model Performance and Temporal Validation

In the development cohort, apparent AUCs were 0.798 (95% CI, 0.759–0.837), 0.846 (95% CI, 0.811–0.881), and 0.875 (95% CI, 0.843–0.907) for Models 1, 2, and 3, respectively. Adding hematoma volume and anatomical extension variables to the clinical model increased the AUC by 0.048 (DeLong test, *p* < 0.001) and improved model fit (likelihood ratio test, *p* < 0.001). Adding absolute MLS increased the AUC by a further 0.029 (DeLong test, *p* = 0.014); Model 3 also showed better fit than Model 2 (likelihood ratio test, *p* < 0.001). Bootstrap internal validation yielded optimism-corrected AUCs of 0.773, 0.832, and 0.856 for Models 1, 2, and 3, respectively. The ranking of models by discrimination was unchanged after correction. The equations derived in the development cohort were applied to the temporal validation cohort without coefficient re-estimation or recalibration. In this cohort, AUCs were 0.772 (95% CI, 0.716–0.828), 0.829 (95% CI, 0.779–0.879), and 0.854 (95% CI, 0.807–0.901) for Models 1, 2, and 3, respectively, preserving the same ranking. The calibration curve for Model 3 showed generally good agreement between predicted probabilities and observed outcomes. Figure 3 presents the apparent receiver operating characteristic (ROC) curves for the development cohort and the ROC curves for the temporal validation cohort, together with Model 3 calibration plots with bootstrap 95% confidence intervals, calibration measures, and Brier scores for both cohorts.

### 3.5. Secondary Analysis of the MLS/ICH Volume Ratio

In the secondary analysis, the MLS/ICH volume ratio was added to Model 2 as an alternative to absolute MLS. Each 0.1 mm/mL increase in the ratio was independently associated with 90-day poor functional outcome after adjustment for the other model variables (adjusted OR, 1.30; 95% CI, 1.10–1.54; *p* = 0.002). The secondary model had an AUC of 0.86 (95% CI, 0.83–0.89) in the development cohort and 0.84 (95% CI, 0.79–0.89) in the temporal validation cohort.

### 3.6. Sensitivity Analysis

After excluding 72 patients from the development cohort and 26 from the temporal validation cohort whose 90-day mRS scores had been assigned retrospectively from follow-up records, the sensitivity analysis included 691 patients: 436 in the development cohort and 255 in the temporal validation cohort. The ranking of models by discrimination and the effect size for MLS were similar to those in the primary analysis. MLS remained independently associated with poor functional outcome in separate analyses excluding patients who underwent surgery or EVD and patients with documented care limitations or indeterminate care-limitation status. The association also remained statistically significant after the combined exclusions, although the point estimate was smaller than in the primary analysis (adjusted OR per 1 mm increase, 1.11; 95% CI, 1.006–1.224; *p* = 0.037). After adjustment for onset-to-CT time, MLS remained associated with poor functional outcome (adjusted OR, 1.16; 95% CI, 1.062–1.267; *p* = 0.001). Model 3 had numerically higher AUCs than Model 2 in both cohorts across all sensitivity analyses, with smaller differences after the combined exclusions. Sample sizes, poor outcome counts, AUCs with confidence intervals, and adjusted associations for MLS are presented in Table 4.

## 4. Discussion

In this study, combining radiological features reflecting hematoma volume, anatomical extension, and mass effect with clinical information improved discrimination for 90-day poor functional outcome in the development cohort. Adding hematoma volume, deep structure involvement, and IVH to the clinical model increased the AUC from 0.798 to 0.846. The addition of MLS increased the AUC to 0.875. Hematoma volume, IVH, and MLS remained associated with poor functional outcome in the final model, whereas deep structure involvement showed no significant independent association. Model 3 also showed the highest discrimination in temporal validation, with an AUC of 0.854 when applied without changing its coefficients.

Larger hematoma volume remained associated with 90-day poor functional outcome after accounting for clinical characteristics, intraventricular extension, and MLS. A multicenter study reported that the association between hematoma expansion and 90-day poor outcome varied according to the final hematoma volume and hemorrhage location [19]. Although that study examined expansion and final volume rather than baseline volume, its findings support interpreting hematoma burden alongside anatomical location.

IVH also remained associated with poor functional outcome after accounting for clinical characteristics, parenchymal hematoma volume, and MLS. A study assessing intraventricular blood volume found that volumes exceeding 6 mL were independently associated with poor functional outcome at three months, defined as mRS ≥ 3 [20]. Although its outcome definition differed from ours, this finding suggests that the extent of ventricular bleeding may also be important. In our analysis, IVH was recorded only as present or absent; therefore, the association between intraventricular blood volume and outcome could not be assessed.

The absence of a significant independent association for deep structure involvement should be interpreted in the context of the anatomical classification used. A study of location-specific volume thresholds found that smaller hematoma volumes were associated with poor functional outcome at six months in the thalamus and internal capsule/globus pallidus regions than in lobar or putamen/external capsule locations [21]. Grouping basal ganglia and thalamic involvement together, regardless of accompanying lobar extension, may have obscured regional differences in our study. The finding should therefore not be interpreted as evidence that anatomical location lacks prognostic relevance.

Hematoma volume describes the amount of bleeding, whereas MLS is one radiological indicator of hemorrhage-related mass effect [15]. In a study of patients who underwent CT within the first six hours, the maximum MLS measured at different anatomical levels was associated with poor functional outcome at three months after accounting for admission GCS score, hematoma volume, and other prognostic characteristics [13]. Another study associated MLS greater than 3 mm on baseline CT with poor functional outcome at three months, defined as mRS ≥ 3, and developed a prediction model for this outcome using clinical and imaging features [14].

A multicenter study also combined imaging findings, including hematoma volume, location, shape, density characteristics, and MLS, with clinical information to compare models predicting discharge outcomes [22]. Our study extends this work by assessing the incremental contribution of MLS in sequential models for 90-day functional outcome, with temporal validation. Its contribution after accounting for hematoma volume and anatomical extension suggests that displacement provides information beyond the amount and distribution of bleeding.

In the secondary analysis, the MLS/ICH volume ratio was also associated with poor functional outcome. A high ratio may reflect pronounced MLS or a small hematoma, so it should be interpreted alongside both absolute measurements. Replacing absolute MLS with the ratio while retaining hematoma volume yielded numerically lower AUCs in both cohorts. These differences do not establish model superiority, but the findings provide insufficient evidence to favor the ratio over absolute MLS.

Prediction models should be evaluated in new patient groups using both discrimination and calibration [23]. In our study, Model 3 had a bootstrap optimism-corrected AUC of 0.856. Predicted risks also showed general agreement with observed outcomes in temporal validation.

Approximately 30% of patients in each cohort underwent surgery or EVD. MLS remained associated with poor functional outcome after excluding these patients and, separately, those with documented or indeterminate care limitations. The association persisted after the combined exclusions, although its point estimate and the numerical AUC difference between Models 3 and 2 were smaller. Thus, the association was also present among conservatively managed patients without documented care limitations. These analyses address the influence of these patient groups but do not estimate treatment effects.

Patients with poor outcomes had shorter onset-to-CT intervals in both cohorts, possibly reflecting earlier presentation or assessment in those with more severe symptoms. After adjustment for CT timing, MLS remained associated with poor outcome, and Model 3 retained numerically higher AUCs than Model 2. This supports the consistency of the findings after accounting for differences in the timing of the initial scan.

The use of routine admission data and baseline CT measurements is a practical feature of the model for early prognostic assessment. The estimated risks reflect outcomes under the care provided at our center. The 2022 AHA/ASA ICH guideline likewise states that prognostic scores can help assess disease severity but should not be the sole basis for individual prognostication or decisions to limit life-sustaining treatment [5]. Whether using this model alongside neurological examination and assessment of the clinical course improves prognostic discussions with families, clinical decisions, or patient outcomes requires prospective evaluation.

Strengths of this study include bootstrap internal validation and application of the developed models to a temporal validation cohort without changing their coefficients. Radiological measurements were performed blinded to clinical outcomes, helping to reduce assessment bias. The preservation of model ranking and the association between MLS and poor outcome after excluding patients whose 90-day mRS scores were derived from follow-up records further supports the consistency of the main findings.

The single-center retrospective design introduces the possibility of selection and information bias. Restricting inclusion to patients with CT at our center within six hours and a GCS score recorded before sedation and intubation limits generalizability. Excluded patients were more frequently transferred from another institution or intubated before arrival and had lower GCS scores when assessments were available. Their 30-day mortality was also higher in the development period. These differences suggest that the study population may underrepresent patients with more severe presentations. Although missing data were uncommon, complete-case analysis may have introduced bias if patients excluded because of missing data differed systematically from those included. Retrospective mRS assignment may also have introduced misclassification that the sensitivity analysis cannot exclude. The absence of preadmission functional status from the model limited assessment of the association between pre-existing disability and 90-day outcome.

Excluding patients according to treatment and care limitations changes the patient groups being evaluated and cannot fully address treatment-selection bias. Care-limitation assessment depended on documentation and the first recorded decision, potentially missing later changes in care; timing was unavailable in some patients. The low proportion of patients with documented care limitations relative to 30-day mortality suggests that these decisions may have been under-recorded. Some patients without documented limitations may therefore have received limited treatment, which could have affected the sensitivity analyses. Resuscitation decisions also differ in scope from withholding or withdrawing other life-sustaining treatments. Their combined exclusion should not be taken to imply equivalent effects on outcome.

Imaging analysis was limited to baseline CT, so hematoma expansion and subsequent changes in MLS or edema could not be assessed or accounted for by adjusting for CT timing. Estimating hematoma volume with the ABC/2 method may introduce measurement error, particularly in irregularly shaped hemorrhages [24]. Because interobserver agreement was not assessed, the reproducibility of radiological measurements across evaluators remains uncertain. The lack of separate assessments of edema volume, intracranial pressure, and hydrocephalus also limits mechanistic interpretation of the findings related to mass effect and ventricular extension. Incomplete vascular imaging may have left secondary causes undetected, particularly in patients who died before the etiological work-up was completed. Furthermore, no direct comparison with established prognostic scores was performed, so superiority over these approaches cannot be inferred. As temporal validation was conducted at the same center, the model’s performance in different patient populations and care settings requires validation at independent centers.

## 5. Conclusions

In patients with spontaneous supratentorial ICH, adding MLS to a model containing clinical characteristics, hematoma volume, and anatomical extension improved discrimination for 90-day poor functional outcome in the development cohort. Together with the temporal validation findings from the same center, these results suggest that hematoma volume and mass effect may provide complementary prognostic information. Validation at other centers and evaluation of the approach’s contribution to clinical decision-making are needed.

## Figures and Tables

**Figure 1:**
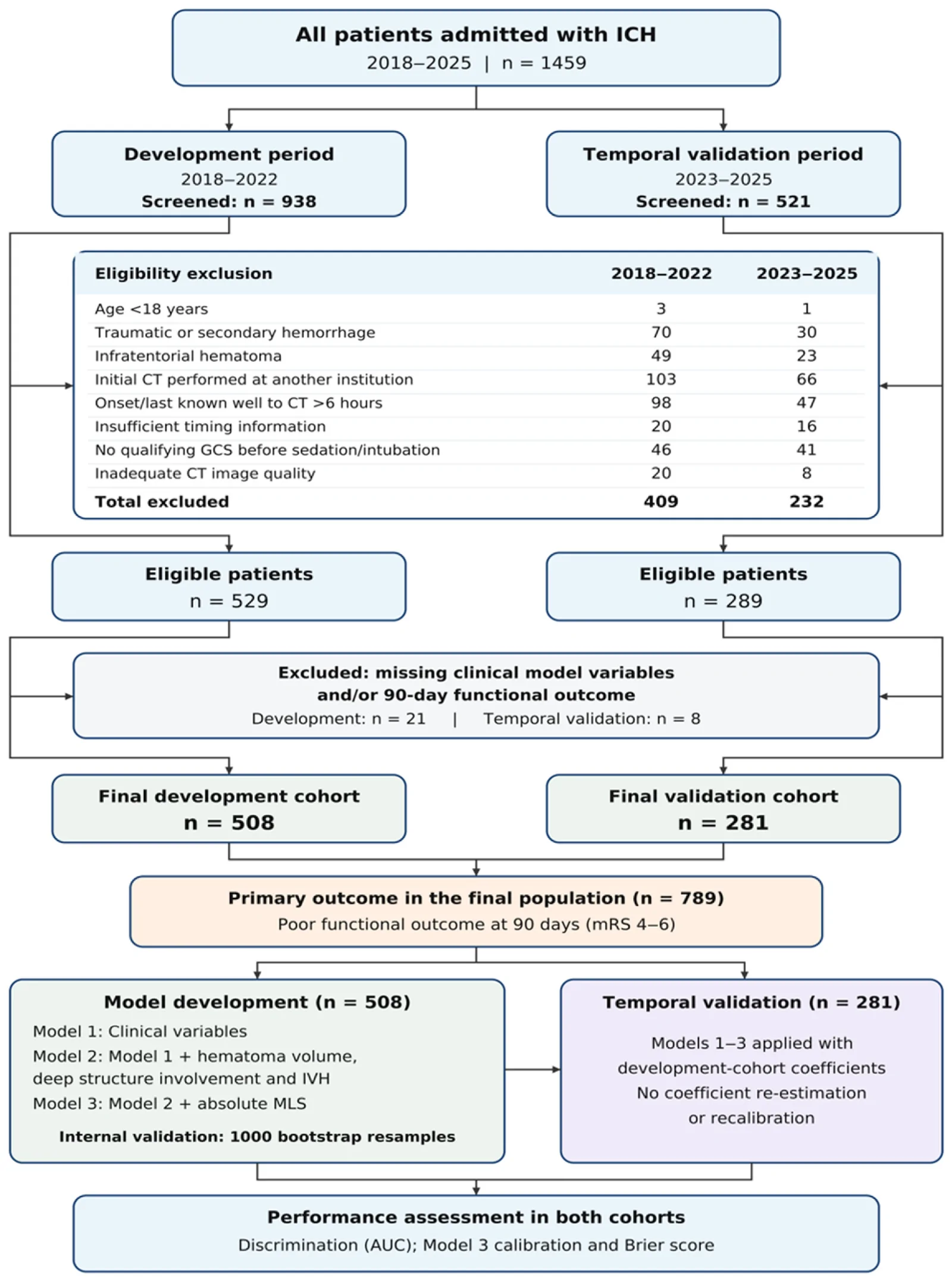
Schematic overview of the study workflow. The diagram summarizes patient selection, exclusions by reason, cohort formation, and prognostic model development and validation. Each excluded patient was counted once under the first applicable criterion. AUC: area under the receiver operating characteristic curve; CT, computed tomography; GCS, Glasgow Coma Scale; ICH, intracerebral hemorrhage; IVH, intraventricular hemorrhage; MLS, midline shift; mRS, modified Rankin Scale.

**Figure 2:**
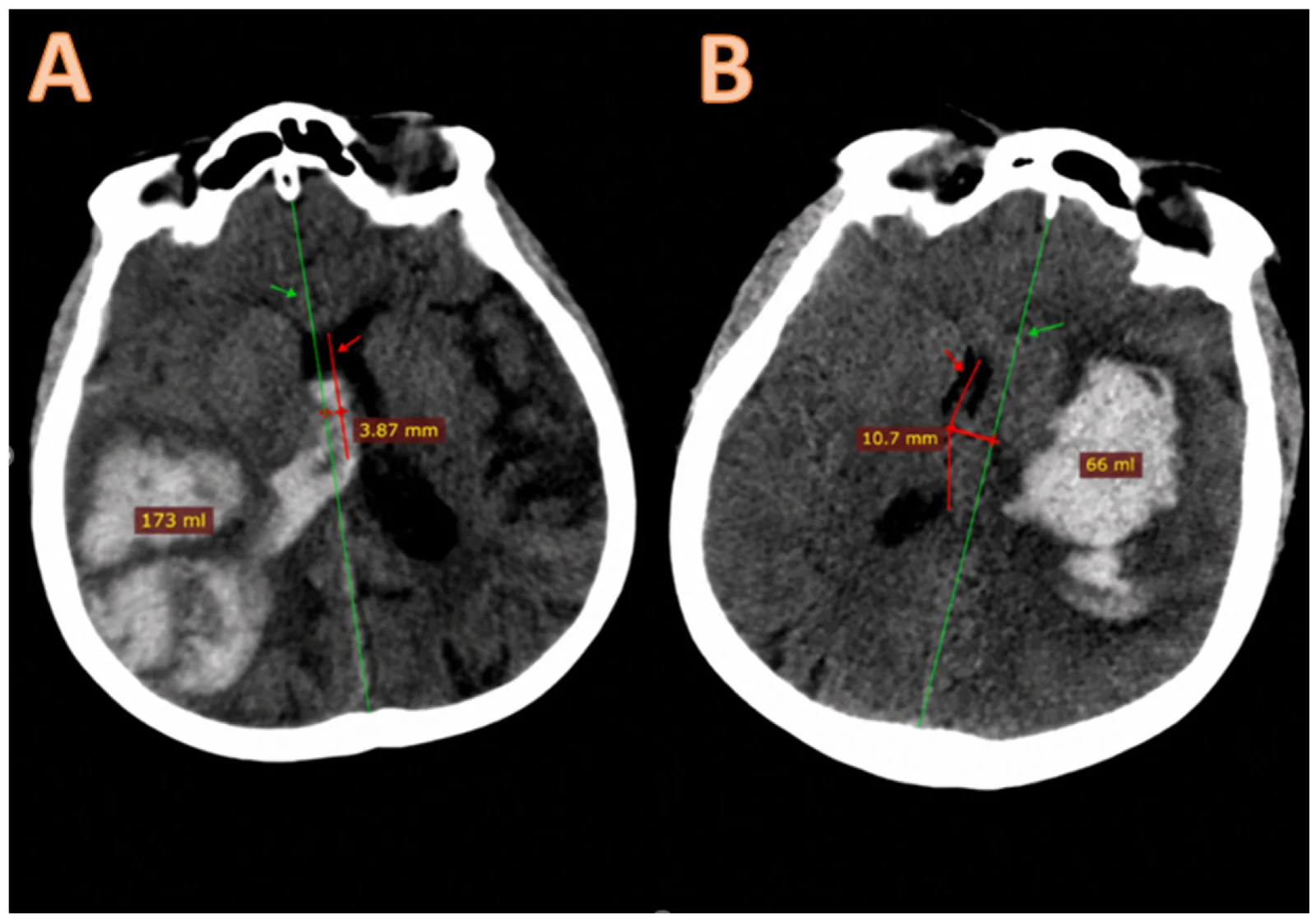
Representative non-contrast axial CT images showing different hematoma volumes and degrees of midline shift (MLS) in two patients with spontaneous supratentorial intracerebral hemorrhage. (**A**) Larger hematoma with less pronounced MLS. (**B**) Relatively smaller hematoma with more pronounced MLS. Green arrows and lines indicate the anatomical midline; red arrows and lines indicate the septum pellucidum. MLS was measured as the perpendicular distance between the anatomical midline and the displaced septum pellucidum. Hematoma volumes were estimated using the ABC/2 method across the full CT series.

**Figure 3:**
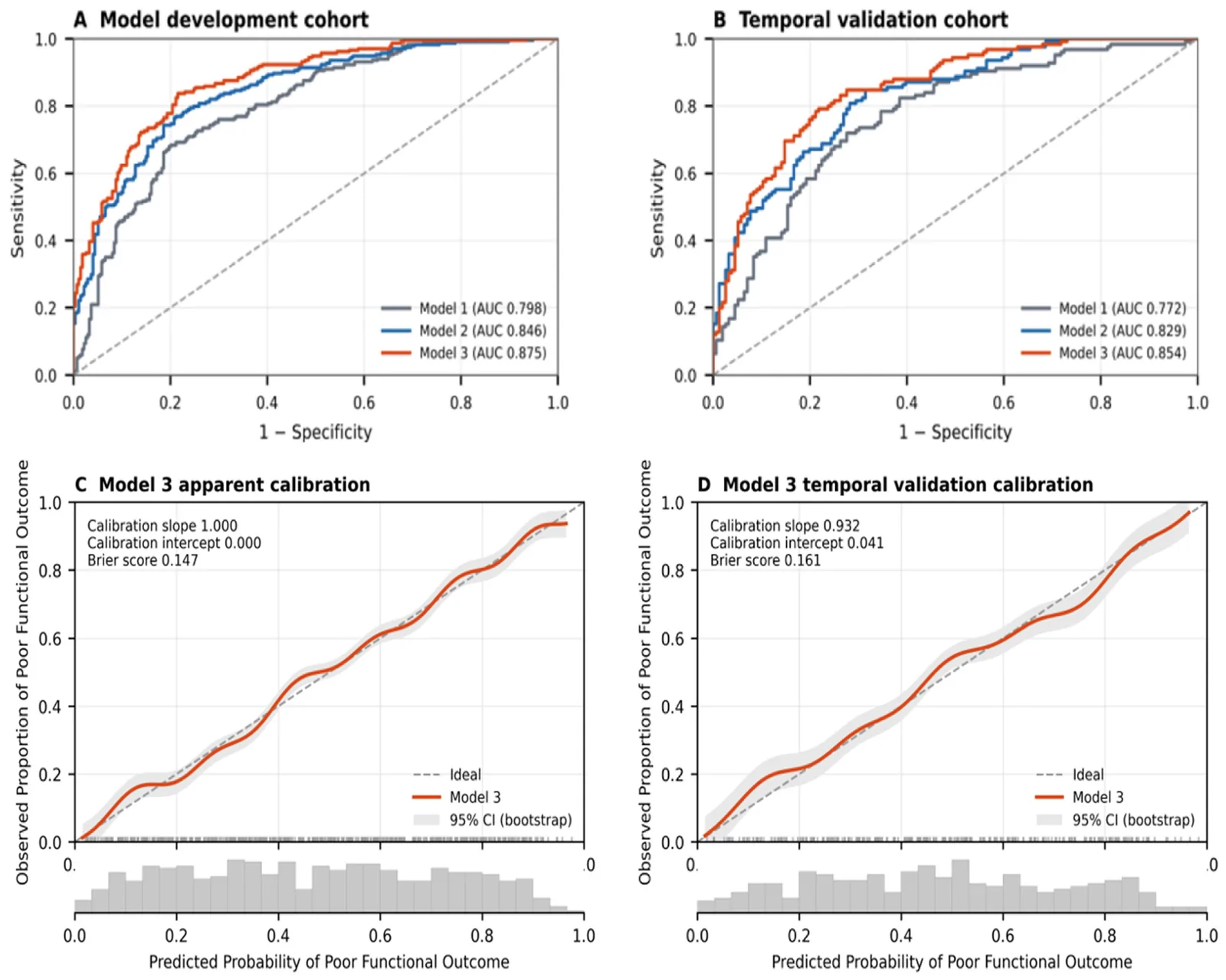
Discrimination and calibration performance of the sequential prognostic models. Receiver operating characteristic curves for Models 1–3 in the model development cohort (**A**) and temporal validation cohort (**B**). Calibration plots of the final Model 3 in the model development cohort (**C**) and temporal validation cohort (**D**). Model 1 included clinical variables; Model 2 additionally included hematoma volume, deep structure involvement, and intraventricular hemorrhage; and Model 3 further included absolute midline shift. The diagonal dashed lines indicate no discrimination in the ROC panels and perfect agreement between predicted and observed probabilities in the calibration panels. In panels (**C**,**D**), solid lines represent smoothed calibration curves, shaded areas represent bootstrap 95% confidence intervals, rug marks indicate individual predicted probabilities, and the lower histograms show the distributions of predicted probabilities. ROC, receiver operating characteristic; AUC, area under the receiver operating characteristic curve.

**Table 1:** Demographic, clinical, radiological, treatment, and outcome characteristics of the study cohorts; comparisons between included and excluded patients.

Panel A. Characteristics of the study cohorts					
Characteristic		Model Development Cohort (n = 508)		Temporal Validation Cohort (n = 281)	
Demographic characteristics					
Age, years		64 (55–73)		65 (56–74)	
Male sex, n (%)		297 (58.5)		160 (56.9)	
Clinical characteristics at admission					
Glasgow Coma Scale score		11 (8–14)		11 (8–15)	
Systolic blood pressure, mmHg		176 ± 31		173 ± 31	
Serum glucose, mg/dL		148 (118–192)		145 (116–188)	
INR		1.08 (0.99–1.24)		1.07 (0.98–1.21)	
Preadmission anticoagulant use, n (%)		74 (14.6)		38 (13.5)	
CT timing					
Onset-to-CT interval, minutes		85 (55–125)		80 (50–122.5)	
Last known well time used, n (%)		41 (8.1)		24 (8.5)	
Radiological characteristics					
Hematoma volume, mL		24 (13–41)		23 (12–39)	
Midline shift, mm		4.0 (2.0–7.0)		4.0 (2.0–6.0)	
Deep structure involvement, n (%)		312 (61.4)		168 (59.8)	
Intraventricular hemorrhage, n (%)		196 (38.6)		103 (36.7)	
MLS/ICH volume ratio, mm/mL		0.17 (0.09–0.31)		0.16 (0.08–0.29)	
Vascular imaging during hospitalization					
Any vascular imaging, n (%)		354 (69.7)		216 (76.9)	
CT angiography, n (%)		338 (66.5)		208 (74.0)	
Magnetic resonance angiography, n (%)		42 (8.3)		24 (8.5)	
Digital subtraction angiography, n (%)		30 (5.9)		20 (7.1)	
Treatment during hospitalization					
Any surgery or EVD, n (%)		150 (29.5)		82 (29.2)	
Craniotomy for hematoma evacuation, n (%)		80 (15.7)		40 (14.2)	
Decompressive craniectomy with or without hematoma evacuation, n (%)		30 (5.9)		20 (7.1)	
Minimally invasive hematoma evacuation, n (%)		10 (2.0)		6 (2.1)	
EVD, n (%)		60 (11.8)		36 (12.8)	
Conservative management, n (%)		358 (70.5)		199 (70.8)	
Limitation-of-care status					
Documented limitation, n (%)		12 (2.4)		6 (2.1)	
No documented limitation, n (%)		472 (92.9)		265 (94.3)	
Indeterminate status, n (%)		24 (4.7)		10 (3.6)	
Type of first documented care-limitation decision					
Do-not-resuscitate decision, n (%)		7 (1.4)		3 (1.1)	
Withholding or non-escalation of life-sustaining treatment, n (%)		4 (0.8)		2 (0.7)	
Withdrawal of life-sustaining treatment, n (%)		1 (0.2)		1 (0.4)	
Time from admission to first care-limitation decision					
≤24 hours, n (%)		3 (0.6)		1 (0.4)	
>24–48 hours, n (%)		2 (0.4)		1 (0.4)	
>48 hours, n (%)		5 (1.0)		3 (1.1)	
Timing unavailable, n (%)		2 (0.4)		1 (0.4)	
Clinical outcomes					
Poor functional outcome at 90 days, n (%)		234 (46.1)		125 (44.5)	
30-day mortality, n (%)		126 (24.8)		65 (23.1)	
Panel B. Comparisons between included and excluded patients					
Model development period					
Characteristic		Included (n = 508)	Excluded (n = 430)		*p*-Value
Age, years		64 (55–73)	66 (54–77)		0.069
Male sex, n (%)		297 (58.5)	263 (61.2)		0.401
Transferred from another institution, n (%)		66 (13.0)	161 (37.4)		<0.001
Intubated before arrival, n (%)		0 (0.0)	79 (18.4)		<0.001
Pre-sedation GCS available, n (%)		508 (100.0)	273 (63.5)		<0.001
Glasgow Coma Scale score		11 (8–14)	9 (7–12)		<0.001
30-day mortality, n (%)		126 (24.8)	141 (32.8)		0.007
Temporal validation period					
Characteristic		Included (n = 281)	Excluded (n = 240)		*p*-Value
Age, years		65 (56–74)	67 (56–78)		0.523
Male sex, n (%)		160 (56.9)	138 (57.5)		0.897
Transferred from another institution, n (%)		24 (8.5)	97 (40.4)		<0.001
Intubated before arrival, n (%)		0 (0.0)	37 (15.4)		<0.001
Pre-sedation GCS available, n (%)		281 (100.0)	161 (67.1)		<0.001
Glasgow Coma Scale score		11 (8–15)	10 (7–12)		<0.001
30-day mortality, n (%)		65 (23.1)	64 (26.7)		0.351

Values are presented as median (interquartile range), mean ± standard deviation, or number (%). Poor functional outcome was defined as mRS 4–6. Percentages use the corresponding cohort or comparison-group denominator. Imaging and procedure categories may overlap; patients were counted once in each “any” category. Conservative management indicates no listed procedure. Care-limitation status categories are mutually exclusive; indeterminate status reflects insufficient documentation. Decision type and timing refer to the first documented care limitation. In Panel B, excluded patients include those excluded for eligibility criteria or missing model variables or outcome data. GCS summaries use available assessments (508 versus 273 patients in the development period; 281 versus 161 in the validation period). Comparisons used the Mann–Whitney U test or Pearson’s chi-square test, as appropriate. ICH, intracerebral hemorrhage; INR, international normalized ratio; MLS, midline shift; mRS, modified Rankin Scale; CT, computed tomography; EVD, external ventricular drainage; GCS, Glasgow Coma Scale.

**Table 2:** Clinical and radiological characteristics according to 90-day functional outcome in the model development and temporal validation cohorts.

Panel A. Model Development Cohort			
Characteristic	Good Outcome (mRS 0–3; n = 274)	Poor Outcome (mRS 4–6; n = 234)	*p*-Value
Age, years	60 (51–68)	69 (61–77)	<0.001
Male sex, n (%)	158 (57.7)	139 (59.4)	0.692
Glasgow Coma Scale score	14 (11–15)	8 (6–11)	<0.001
Systolic blood pressure, mmHg	174 ± 29	178 ± 33	0.146
Serum glucose, mg/dL	132 (108–165)	171 (132–224)	<0.001
INR	1.06 (0.98–1.19)	1.10 (1.00–1.29)	0.073
Preadmission anticoagulant use, n (%)	36 (13.1)	38 (16.2)	0.323
Onset-to-CT interval, minutes	96.5 (64.9–164.9)	73.4 (43.2–117.0)	0.023
Hematoma volume, mL	16 (9–27)	36 (22–55)	<0.001
Midline shift, mm	2.0 (1.0–4.0)	7.0 (4.0–10.0)	<0.001
Deep structure involvement, n (%)	159 (58.0)	153 (65.4)	0.090
Intraventricular hemorrhage, n (%)	69 (25.2)	127 (54.3)	<0.001
MLS/ICH volume ratio, mm/mL	0.11 (0.06–0.22)	0.23 (0.13–0.39)	<0.001
Panel B. Temporal Validation Cohort			
Characteristic	Good Outcome (mRS 0–3; n = 156)	Poor Outcome (mRS 4–6; n = 125)	*p*-Value
Age, years	59 (50–68)	71 (63–79)	<0.001
Male sex, n (%)	87 (55.8)	73 (58.4)	0.658
Glasgow Coma Scale score	13 (10–15)	7 (5–10)	<0.001
Systolic blood pressure, mmHg	169 ± 30	177 ± 32	0.031
Serum glucose, mg/dL	127 (105–158)	174 (134–226)	<0.001
INR	1.04 (0.97–1.17)	1.11 (1.00–1.30)	0.046
Preadmission anticoagulant use, n (%)	19 (12.2)	19 (15.2)	0.462
Onset-to-CT interval, minutes	82.7 (48.5–158.7)	76.6 (51.3–114.6)	0.041
Hematoma volume, mL	14 (7–25)	38 (23–57)	<0.001
Midline shift, mm	2.0 (0.0–4.0)	6.0 (3.0–9.0)	<0.001
Deep structure involvement, n (%)	91 (58.3)	77 (61.6)	0.579
Intraventricular hemorrhage, n (%)	38 (24.4)	65 (52.0)	<0.001
MLS/ICH volume ratio, mm/mL	0.12 (0.05–0.23)	0.20 (0.10–0.35)	<0.001

Values are presented as median (interquartile range), mean ± standard deviation, or number (%), as appropriate. Between-group comparisons used the independent-samples *t*-test or Mann–Whitney U test for continuous variables and Pearson’s chi-square test or Fisher’s exact test for categorical variables, as appropriate. CT, computed tomography; ICH, intracerebral hemorrhage; INR, international normalized ratio; MLS, midline shift; mRS, modified Rankin Scale.

**Table 3:** Multivariable logistic regression models for 90-day poor functional outcome in the model development cohort.

Variable	Model 1		Model 2		Model 3	
	Adjusted OR (95% CI)	*p*-Value	Adjusted OR (95% CI)	*p*-Value	Adjusted OR (95% CI)	*p*-Value
Clinical Characteristics						
Age (per 10-year increase)	1.25 (1.09–1.44)	0.002	1.19 (1.03–1.38)	0.019	1.17 (1.01–1.37)	0.036
Male sex	1.07 (0.75–1.52)	0.708	1.06 (0.74–1.52)	0.744	1.08 (0.76–1.53)	0.667
Glasgow Coma Scale (per 1-point increase)	0.84 (0.80–0.88)	<0.001	0.88 (0.84–0.92)	<0.001	0.89 (0.85–0.93)	<0.001
Systolic blood pressure (per 10-mmHg increase)	1.04 (0.97–1.12)	0.254	1.03 (0.96–1.11)	0.389	1.02 (0.95–1.10)	0.574
Serum glucose (per 10 mg/dL increase)	1.042 (1.012–1.073)	0.005	1.032 (1.003–1.062)	0.030	1.029 (1.001–1.058)	0.043
INR (per 0.1-unit increase)	1.02 (0.99–1.05)	0.181	1.01 (0.98–1.04)	0.376	1.01 (0.98–1.04)	0.421
Preadmission anticoagulant use	1.20 (0.73–1.97)	0.469	1.14 (0.70–1.87)	0.602	1.18 (0.72–1.93)	0.512
Hematoma burden and anatomical extension						
Hematoma volume (per 10 mL increase)	—	—	1.28 (1.17–1.40)	<0.001	1.22 (1.10–1.35)	<0.001
Deep structure involvement	—	—	1.12 (0.78–1.61)	0.538	1.09 (0.76–1.56)	0.638
Intraventricular hemorrhage	—	—	1.82 (1.25–2.65)	0.002	1.69 (1.16–2.47)	0.006
Mass effect						
Midline shift (per 1 mm increase)	—	—	—	—	1.17 (1.08–1.28)	<0.001

Model 1 included clinical variables. Model 2 added hematoma volume, deep structure involvement, and IVH to Model 1; Model 3 added absolute MLS to Model 2. An em dash (—) indicates a variable not included in the model. For Model 3, the probability of poor 90-day functional outcome (mRS 4–6) was calculated as P = 1/[1 + exp(−Z)], where Z = −1.92 + 0.1570 × (age/10) + 0.0770 × male sex − 0.1165 × GCS + 0.0198 × (SBP/10) + 0.0286 × (glucose/10) + 0.0100 × (INR/0.1) + 0.1655 × anticoagulant use + 0.1989 × (hematoma volume/10) + 0.0862 × deep structure involvement + 0.5247 × IVH + 0.1570 × MLS. Units: age, years; SBP, mmHg; glucose, mg/dL; hematoma volume, mL; MLS, mm. Male sex is coded as 1 (female = 0); preadmission anticoagulant use, deep structure involvement, and IVH are coded as 1 if present and 0 if absent. OR, odds ratio; CI, confidence interval; GCS, Glasgow Coma Scale; SBP, systolic blood pressure; INR, international normalized ratio; IVH, intraventricular hemorrhage; MLS, midline shift; mRS, modified Rankin Scale.

**Table 4:** Model discrimination and adjusted associations of MLS with 90-day poor functional outcome in sensitivity analyses.

Sensitivity Analysis	Cohort	n/ Poor Outcome, n (%)	Model 1 AUC (95% CI)	Model 2 AUC (95% CI)	Model 3 AUC (95% CI) ΔAUC ^1^	MLS Adjusted OR (95% CI); *p* ^2^
Excluding record-based mRS assessments	Dev.	436 208 (47.7)	0.810 (0.769–0.851)	0.860 (0.824–0.896)	0.890 (0.858–0.922) Δ = 0.030	1.18 (1.08–1.29) *p* <0.001
	Val.	255 116 (45.5)	0.790 (0.733–0.847)	0.840 (0.789–0.891)	0.870 (0.824–0.916) Δ = 0.030	—
Excluding surgery or EVD	Dev.	358 115 (32.1)	0.782 (0.727–0.837)	0.826 (0.776–0.876)	0.849 (0.802–0.896) Δ = 0.023	1.14 (1.024–1.270) *p* = 0.017
	Val.	199 63 (31.7)	0.759 (0.683–0.835)	0.803 (0.732–0.874)	0.821 (0.752–0.890) Δ = 0.018	—
Excluding documented care limitations or indeterminate status	Dev.	472 209 (44.3)	0.793 (0.751–0.835)	0.841 (0.804–0.878)	0.869 (0.835–0.903) Δ = 0.028	1.16 (1.052–1.279) *p* = 0.003
	Val.	265 113 (42.6)	0.768 (0.709–0.827)	0.824 (0.771–0.877)	0.846 (0.796–0.896) Δ = 0.022	—
Combined exclusions	Dev.	340 108 (31.8)	0.775 (0.718–0.832)	0.818 (0.765–0.871)	0.835 (0.784–0.886) Δ = 0.017	1.11 (1.006–1.224) *p* = 0.037
	Val.	191 59 (30.9)	0.752 (0.672–0.832)	0.793 (0.718–0.868)	0.807 (0.734–0.880) Δ = 0.014	—
Adjustment for onset-to-CT time	Dev.	508 234 (46.1)	0.805 (0.766–0.844)	0.849 (0.814–0.884)	0.877 (0.845–0.909) Δ = 0.028	1.16 (1.062–1.267) *p* = 0.001
	Val.	281 125 (44.5)	0.780 (0.724–0.836)	0.832 (0.782–0.882)	0.855 (0.809–0.901) Δ = 0.023	—

Poor outcome was defined as mRS 4–6; percentages were calculated within each analysis subset. Combined exclusions comprised surgery or EVD, documented care limitations, and indeterminate care-limitation status. ^1^ ΔAUC = Model 3 AUC—Model 2 AUC, calculated from the displayed AUCs. ^2^ Adjusted ORs and *p*-values refer to MLS in Model 3 of the development cohort, per 1 mm increase; *p*-values do not refer to AUC comparisons. —, not applicable: development-cohort coefficients were applied unchanged in temporal validation. Dev., development; Val., temporal validation; AUC, area under the receiver operating characteristic curve; CI, confidence interval; CT, computed tomography; EVD, external ventricular drainage; MLS, midline shift; mRS, modified Rankin Scale; OR, odds ratio.

## Data Availability

The data supporting the findings of this study are available from the corresponding author upon reasonable request, subject to institutional approval. The data are not publicly available because of patient confidentiality and institutional restrictions.

## Abbreviations

AHA	American Heart Association
ASA	American Stroke Association
AUC	Area under the curve
CT	Computed tomography
GCS	Glasgow Coma Scale
ICH	Intracerebral hemorrhage
IVH	Intraventricular hemorrhage
MLS	Midline shift
mRS	Modified Rankin Scale
ROC	Receiver operating characteristic
